# Replicative senescent human cells possess altered circadian clocks with a prolonged period and delayed peak-time

**DOI:** 10.18632/aging.101794

**Published:** 2019-02-09

**Authors:** Rezwana Ahmed, Atsushige Ashimori, Satoshi Iwamoto, Takaaki Matsui, Yasukazu Nakahata, Yasumasa Bessho

**Affiliations:** 1 Laboratory of Gene Regulation Research, Division of Biological Science, Graduate School of Science and Technology, Nara Institute of Science and Technology (NAIST), Ikoma, Nara 630-0192, Japan

**Keywords:** circadian clock, cellular senescence, aging, primary fibroblast, TIG-3 cells

## Abstract

Over the last decade, a wide array of evidence has been accumulated that disruption of circadian clock is prone to cause age-related diseases and premature aging. On the other hand, aging has been identified as one of the risk factors linked to the alteration of circadian clock. These evidences suggest that the processes of aging and circadian clock feedback on each other at the animal level. However, at the cellular level, we recently revealed that the primary fibroblast cells derived from *Bmal1^-/-^* mouse embryo, in which circadian clock is completely disrupted, do not demonstrate the acceleration of cellular aging, i.e., cellular senescence. In addition, little is known about the impact of cellular senescence on circadian clock. In this study, we show for the first time that senescent cells possess a longer circadian period with delayed peak-time and that the variability in peak-time is wider in the senescent cells compared to their proliferative counterparts, indicating that senescent cells show alterations of circadian clock. We, furthermore, propose that investigation at cellular level is a powerful and useful approach to dissect molecular mechanisms of aging in the circadian clock.

## Introduction

Cellular senescence is a process that imposes permanent proliferative arrest in response to various stresses [1,2]. A wide array of evidence suggests that *in vivo* cellular senescence causes a loss of tissue stem/progenitor cells, and extracellular environment and cells surrounding senescent cells could be functionally disrupted by inflammatory cytokines, growth factors and proteases, which are secreted by senescent cells. In addition, senescent cells have been found at sites of age-related pathologies e.g. atherosclerosis and osteoarthritis [3]. Recent studies demonstrated that pharmacological or genetic eliminations of senescent cells from aging mice extend median lifespan, delayed tumorigenesis and attenuated progression of already established age-related disorders [4–10]. These findings strongly suggest that senescent cells play a key role in these pathological conditions and hence cellular senescence has been termed as the basic driver of the aging phenotype [1]. Despite these clear negative implications, one system on which the impacts of cellular senescence still remain unexplored is the circadian clock.

The circadian clock is driven by the circadian clock gene expressions with approximately a 24-hr rhythm. In mammals, a master clock resides in the hypothalamic suprachiasmatic nucleus (SCN) [11,12]. In contrast, peripheral clocks are distributed among most of the peripheral tissues and even in cultured cells. Both the master and peripheral clocks are controlled by various stimuli to adapt to environmental rhythms generated by the earth’s rotation [13–15]. Malfunction of circadian clock not only disrupts sleep/wake cycles, but also brings about many physiological abnormalities, leading to a wide variety of age-related diseases and premature aging in mice and humans [16–21]. One example is that *Bmal1^-/-^* mice accelerates premature aging accompanied with short lifespan [20]. On the other hand, several studies from model animals and humans have demonstrated that aging can also lead to alteration of the circadian clock [22,23]. Circadian free running periods and days required to re-entrain following new light-dark schedule have been shown to be altered by aging [24–28]. These reports clearly demonstrate that the mechanisms of circadian clock and aging mutually regulate each other at the animal levels.

Although evaluations of impact of aging on the circadian clock *in vivo* provide valuable information and exceedingly contribute to the progress of circadian biology, it is still unclear how the molecular mechanisms of aging affect the circadian clock and *vice versa*. *In vitro* evaluations could have advantages to address that, however, in contrast to various *in vivo* studies few researches at the cellular level have been performed. We have recently reported that primary fibroblast cells derived from *Bmal1^-/-^* mouse embryo, in which circadian clock is completely disrupted, do not display the acceleration of the process of *in vitro* cellular senescence, suggesting that cell-autonomous circadian clock is not implicated in the process of cellular senescence [29]. On the other hand, Kunieda *et al.* have demonstrated that circadian gene expressions display low amplitude in senescent primary cultured human aortic vascular smooth muscle cells [30]. However, they did not mention about the period length probably due to the sample collections with low time-resolution (4 hr). In this study, we utilize TIG-3 cells, normal primary human diploid fibroblast cells [31], and the real-time luciferase monitoring system with high time-resolution (10 min) [32,33] to address whether cellular senescence alters circadian clock properties. We show for the first time that senescent TIG-3 cells possess a longer circadian period with delayed peak-time compared to their proliferative counterparts. Moreover, we observe that senescent TIG-3 cells have defective entrainment mechanism in the circadian oscillator, presumably through altered *PER1* and *PER2* rapid inductions, causing the delayed peak-time. These findings demonstrate that senescent cells possess the altered circadian clock, at least in TIG-3 cells.

## RESULTS

### Replicative senescence of TIG-3 cells

**Figure 1:**
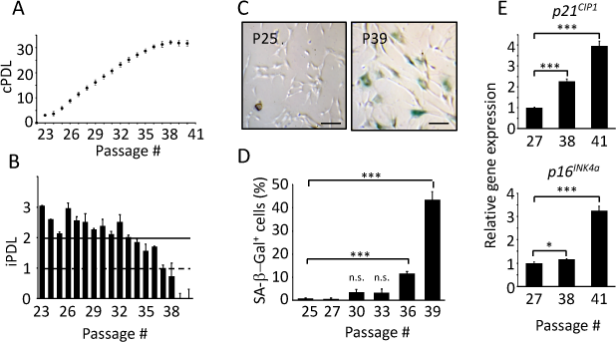
**Replicative senescence of TIG-3 cells.** Cumulative (**A**) and individual (**B**) population doubling level (PDL) of TIG-3 cells were measured. (**C**) Pictures of SA-β-Gal positive cells (blue) at passage25 (P25) and 39 (P39) were taken. Scale bars represent 100 μm. (**D**) The percentage of SA-β-Gal positive cells were quantified at different passages. (**E**) *p21^CIP1^* (upper panel) and *p16^INK4a^* (bottom panel) genes expression at different passages were analyzed by qPCR. The lowest expression level was set to 1. Three independent proliferation assays were performed. n.s., not significant, *p<0.05, ***p<0.001, compared to each lowest passage for (D) and (E), by Student’s two-tailed *t* test.

To evaluate the changes in the circadian clock during senescence, we chose replicative senescence, in which cell cycle would be irreversibly arrested due to the limitation of cell division. TIG-3 cells, which are normal primary diploid human fibroblast cells derived from fetal male lung [31], were serially passaged until they ceased proliferation, that is, reached replicative senescence. When we obtained TIG-3 cells, they had already been passaged 20 times. Therefore, we performed serial passaging starting from Passage 22 (P22). In order to assess at which passage cells begin to cease proliferation, individual and cumulative population doubling level (iPDL and cPDL, respectively) were recorded (Fig.1A and B). Here, we defined a cease of cell proliferation (CCP) as the point at which iPDL value dips below 1.0. Proliferation of TIG-3 cells were fairly constant up to P32, before which iPDLs were above 2. However, iPDLs started to decrease from P33 and TIG-3 cells reached CCP at P37 (Fig.1B).

Senescent cells have unique features, such as enlarged, flattened cell shape, increased senescence-associated β-galactosidase (SA-β-Gal) activity [34,35], and increased expressions of cell cycle inhibitors (*p16^INK4a^, p19^ARF^* and *p21^CIP1^*) [35–37]. We therefore investigated the aforementioned features to confirm that serially passaged TIG-3 cells indeed exhibit senescence features, and to use these features as the basis to group the cells into two categories: proliferative and senescent cells. Representative pictures of SA-β-Gal assay at P25 and P39 are shown in Fig.1C. Senescent cells, which were stained in blue, were observed at P39, in which 43.5% of cells were SA-β-Gal positive, but only 0.8% were positive at P25. Fig.1D represents the percentages of SA-β-Gal positive cells at several passages. SA-β-Gal positive cell number was dramatically increased from P36, at which iPDL was already below 2.0 (Fig.1B), suggesting that decrease in proliferation is due to replicative senescence. Moreover, we observed enlarged, flattened cells at P39 compared to the cells at P25 (Fig.1C), supporting that many cells have already turned senescent at P39. We next measured the expression levels of senescence marker genes, *p21^CIP1^* and *p16^INK4a^*, which are known to initiate and maintain cellular senescence, respectively [38], at P27, P38 and P41. Expression levels of *p21^CIP1^* increased with passages (Fig.1E upper panel). Expression levels of *p16^INK4a^* was high at P41, but not at P27 and P38 (Fig.1E bottom panel). These results indicated that P38 and P41 were at the senescence phase. All the results above indicate that the serially passaged TIG-3 cells indeed reached replicative senescence. Furthermore, we deduced that TIG-3 cells are in the proliferative phase until P32, followed by the transition phase from P33 to P35, and the senescence phase after P36. Hence, in the subsequent parts of this study, TIG-3 cells of P25-29 and of P36-41 were utilized as proliferative and senescent cells, respectively. We performed three independent proliferation assays and could reproduce similar results as shown in Fig.1.

### Senescent TIG-3 cells show altered characteristics of circadian clock

**Figure 2:**
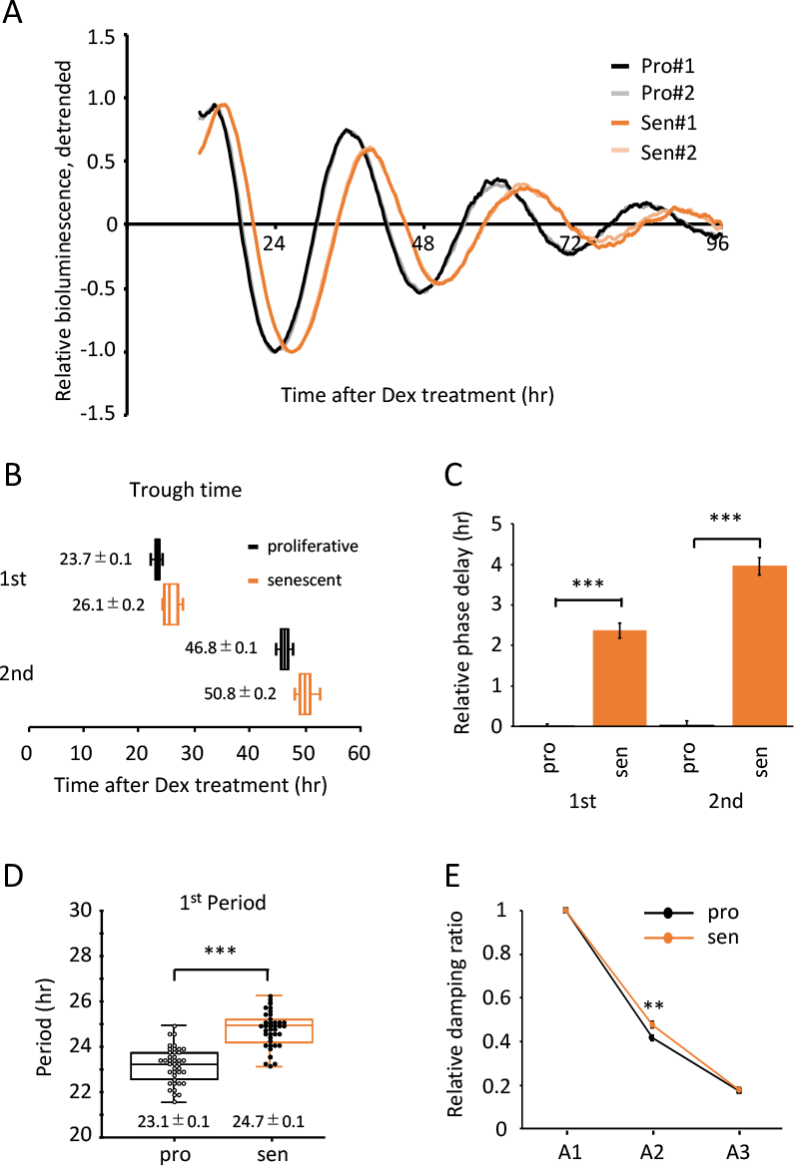
**Alteration of circadian clock in the senescent cells was observed by a Dex-induced entrainment.** (**A**) Relative oscillation patterns of luciferase in the proliferative and senescent cells were monitored by using a real-time luciferase monitoring system. Lowest intensity of each sample was set to -1. (**B**) Box-whisker plots of trough-times are displayed, n=41. Values are mean ± SEM. (**C**) Relative trough-time differences were measured, n=41. (**D**) Box-whisker plots of period lengths in the proliferative (pro) and senescent (sen) cells are displayed, n=41. Values are mean ± SEM. (**E**) Relative damping ratio in the proliferative (pro) and the senescent (sen) cells were analyzed. Data are mean ± SEM. See Fig.S3 for more information. *p<0.05, **p<0.01, ***p<0.001, compared to each of the proliferative cells by Student’s two-tailed *t* test.

To address the changes in the circadian clock during senescence, virus containing *bmal1* promoter-driven luciferase gene [39] was infected to the proliferative and senescent TIG-3 cells obtained from three independent proliferation assays. The infected cells were synchronized with dexamethasone (Dex) and subjected to the real-time luciferase assay. Fig.2A shows the superimposition of relative oscillatory patterns of luciferase (See Fig. S1 for raw data of oscillation patterns). In order to exclude the possibility that the phase and/or luciferase intensity of the first peak are modified by acute effect by Dex treatment, we normalized oscillation patterns using the first trough value. The oscillation pattern in the senescent cells did not overlap with that in the proliferative cells and seemed to be delayed (Fig.2A). To investigate the trough-time difference that occurs between the proliferative and senescent cells, trough-times were extracted from each data. The 1^st^ trough-time of the proliferative and senescent cells were 23.7 ± 0.1 hr and 26.1 ± 0.2 hr, respectively (Fig.2B). The 1^st^ trough-time of the senescent cells was 2.4 ± 0.2 hr delayed with significant difference (Fig.2C). Differences of 2^nd^ trough-time between the proliferative and senescent cells were 4.0 ± 0.2 hr (Fig.2C), indicating that trough-time differences have increased. This result suggests that the period length of circadian clock in the senescent cells is longer compared to that in the proliferative cells. To evaluate the period length of the proliferative and senescent cells, we analyzed the duration between two subsequent trough-times. The 1^st^ period, which is the duration between 1^st^ and 2^nd^ trough-times, was 23.1 ± 0.1 hr in the proliferative cells. In contrast, the 1^st^ period in the senescent cells was significantly longer than that in the proliferative cells (24.7 ± 0.1 hr; *p*-value, 5.35 x 10^-14^; Fig.2D). We also analyzed the peak-time differences and the period using 2^nd^ and 3^rd^ peak-times (Fig.S2). Consistent with trough-time differences, differences of 2^nd^ and 3^rd^ peak-time between the proliferative and senescent cells were 3.6 ± 0.2 and 4.0 ± 0.3 hr, respectively (Fig.S2). Again, the 2^nd^ period, which is the duration between 2^nd^ and 3^rd^ peak-times, in the senescent cells also demonstrated a longer period compared to the proliferative cells (23.6 ± 0.1 hr vs 24.2 ± 0.2 hr; *p*-value, 0.03; Fig.S2).

Since the amplitude of circadian clock has been reported to decline with aging at transcriptional [30,40], neural activity [41] and locomotor activity levels [24–26,28], we proceeded to analyze the range of oscillations in TIG-3 cells. To measure the range of oscillation, the detrended luciferase intensity between top to nadir was determined (Fig.S3). Range of the 1^st^ oscillation (A1) was set to 1 and the damping ratios of A2 and A3 were calculated. We found that, unlike previous reports at the tissue and animal levels, the damping ratios between the proliferative and senescent TIG-3 cells were comparable (Fig.2E).

To prove that period lengths and acrophase are different between the proliferative and senescent cells, we mathematically analyzed the data of Fig.2 using the Cosinor software, provided by Dr. R Refinetti [42]. Period lengths were 24.09 ± 0.20 hr and 25.47 ± 0.28 hr for the proliferative and senescent cells, respectively (*p*-value, 1.56 x 10^-4^; Table 1). The acrophases were -238.71 ± 1.23 degree and -272.54 ± 1.69 degree for the proliferative and senescent cells, respectively (*p*-value, 5.79 x 10^-27^; Table 1), indicating that the phase of senescent cells is significantly delayed compared to that of proliferative cells.

**Table 1:** Cosinor Analysis of Experiments in Figure 2.

	**Period (hr)**	**acrophase (degree)**
**proliferative**	24.09 +/- 0.20	-238.71 +/- 1.23
**senescent**	25.47 +/- 0.28	-272.54 +/- 1.69
***p* value**	1.56 x 10^-4^	5.79 x 10^-27^

Student’s two-tailed *t* test were performed, n=41.

In summary, we revealed that the Dex-synchronized senescent TIG-3 cells display a longer period length and the delay of peak-time compared with the proliferative cells, suggesting that senescent cells possess the altered circadian clock.

### Input pathways are comparable between the proliferative and senescent cells

**Figure 3:**
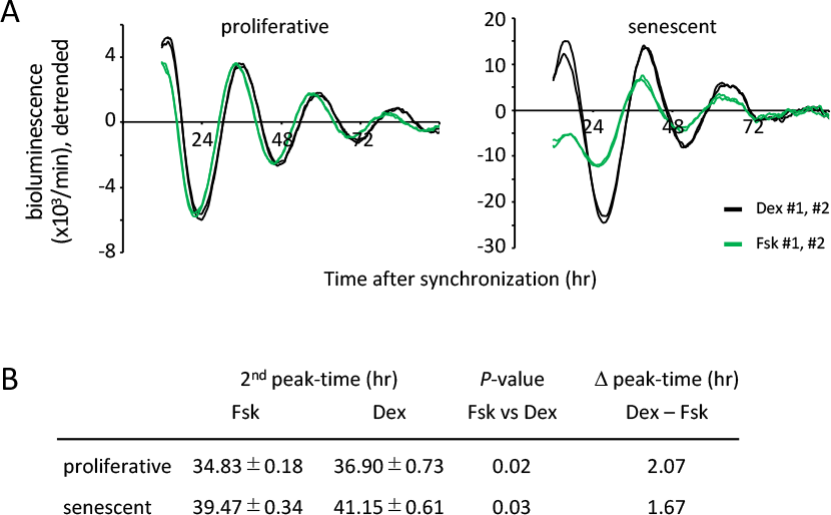
**Input pathways to the oscillator of circadian clocks are comparable between the proliferative and senescent cells.** (**A**) Oscillation patterns of luciferase synchronized by dexamethasone (Dex) or forskolin (Fsk) were monitored by using a real-time luciferase monitoring system. Three independent experiments were performed. (**B**) Summary of peak-times and the difference of peak-time in the proliferative and senescent cells shown in (A). Statistical analyses were performed by Student’s two-tailed *t* test, n=8.

Circadian clock consists of three components; an oscillator with a period of 24 hr, input pathways to the oscillator to allow entrainment of the clock, and output pathways to regulate circadian biochemical, physiological and behavioral rhythms [43,44]. In order to evaluate whether a longer circadian period and delayed peak-time in the senescent TIG-3 cells is the result of the altered oscillator or input pathways, we compared the circadian oscillation patterns of luciferase synchronized by Dex and Forskolin (Fsk). Forskolin is another well-known clock-entrainment factor [14,45,46], activates cAMP-CREB signaling pathway and transcribes *per1* gene via the cAMP response elements (CRE) in *per1* promoter [47]. In contrast, Dex binds to and activates glucocorticoid receptor and transcribes *per1* and *per2* genes via the glucocorticoid response element (GRE) in *per1* and *per2* promoters [48,49]. In the proliferative cells, real-time luciferase monitoring assays demonstrated that circadian oscillation patterns of luciferase activity synchronized by Dex or Fsk show almost similar phase with the same intensity (Fig.3A left panel and Fig.S4). However, consistent with the previous report [46], there was a significant peak-time difference triggered by these two treatments. The second peak of Fsk-treated proliferative cells appeared 2.07 hr earlier than that of Dex-treated proliferative cells (*p*-value, 0.02; Fig.3B). In the senescent cells, the peak-time of the Fsk-treated cells was also significantly earlier compared to the Dex-treated cells (the difference was 1.67 hr; *p*-value, 0.03; Fig.3B). Importantly, the peak-time difference between the proliferative and senescent cells were similar (Fig.3B). Although the first peak and the amplitude of Fsk-treated senescent cells was small and weak, respectively, the above result suggests that both input pathways triggered by Dex or Fsk might not be functionally impaired in senescent cells. Rather, the oscillator of circadian clock was altered with senescence.

### Infection efficiency does not affect the oscillation patterns of luciferase

**Figure 4:**
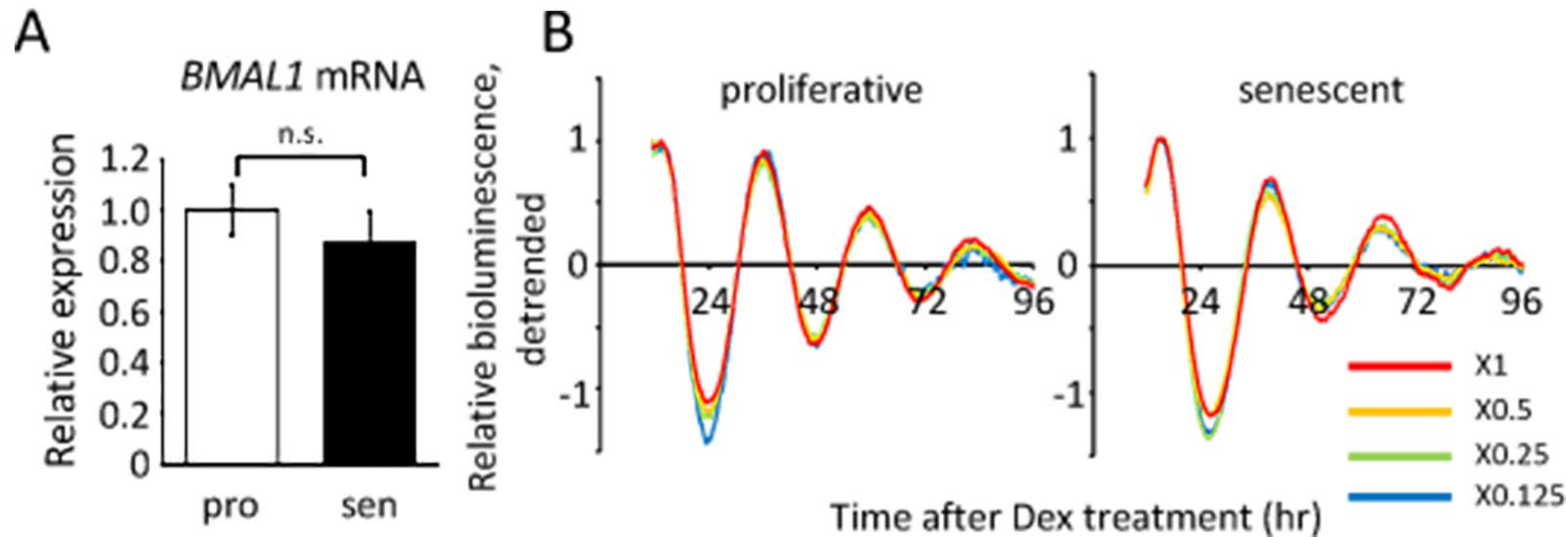
**Infection efficiency does not affect the oscillation patterns of luciferase.** (**A**) *BMAL1* mRNA levels in the proliferative (pro) and senescent (sen) TIG-3 cells were quantified by qPCR. Each sample was normalized by 18S rRNA. *p* = 0.47; n.s., not significant; by Student’s two-tailed *t* test; n=3. Three independent experiments were performed. (**B**) Relative oscillation patterns of luciferase in serially diluted virus-infected TIG-3 cells are shown. Highest intensity of each sample was set to 1. Two independent experiments were performed.

It is also interesting to note that the luciferase activity was higher in the senescent cells than in the proliferative cells (Fig.3A, S1 and S4), giving rise to the possibility that *BMAL1* gene expression is upregulated in the senescent cells, because the *luciferase* gene used in this study was driven by the mouse *bmal1* promoter [39]. To confirm this possibility, we performed qPCR to compare endogenous *BMAL1* transcripts in the non-entrained proliferative and senescent TIG-3 cells. The transcript level of *BMAL1* in the senescent cells was, however, similar or slightly downregulated compared to that in the proliferative cells (Fig.4A). Moreover, circadian expression profile of *BMAL1* in the senescent cells after Dex-entrainment were also similar or lower than those in the proliferative cells (Fig.7D). These results indicate that the high luciferase activity in the senescent cells might not be due to the upregulation of *luciferase* transcription, suggesting that infection efficiency is high in the senescent TIG-3 cells. Therefore, to rule out the possibility that the infection efficiency affects luciferase oscillation patterns, cells were then infected with serially diluted viruses. As expected, luciferase intensities were decreased in a virus-dose dependent manner in both the proliferative and senescent cells (Fig.S5). However, the relative oscillation patterns completely overlapped within the same passages (Fig.4B), demonstrating that the infection efficiency has no effect on the oscillation patterns of luciferase.

### Half-life of luciferase activity is comparable between the proliferative and senescent cells

**Figure 5:**
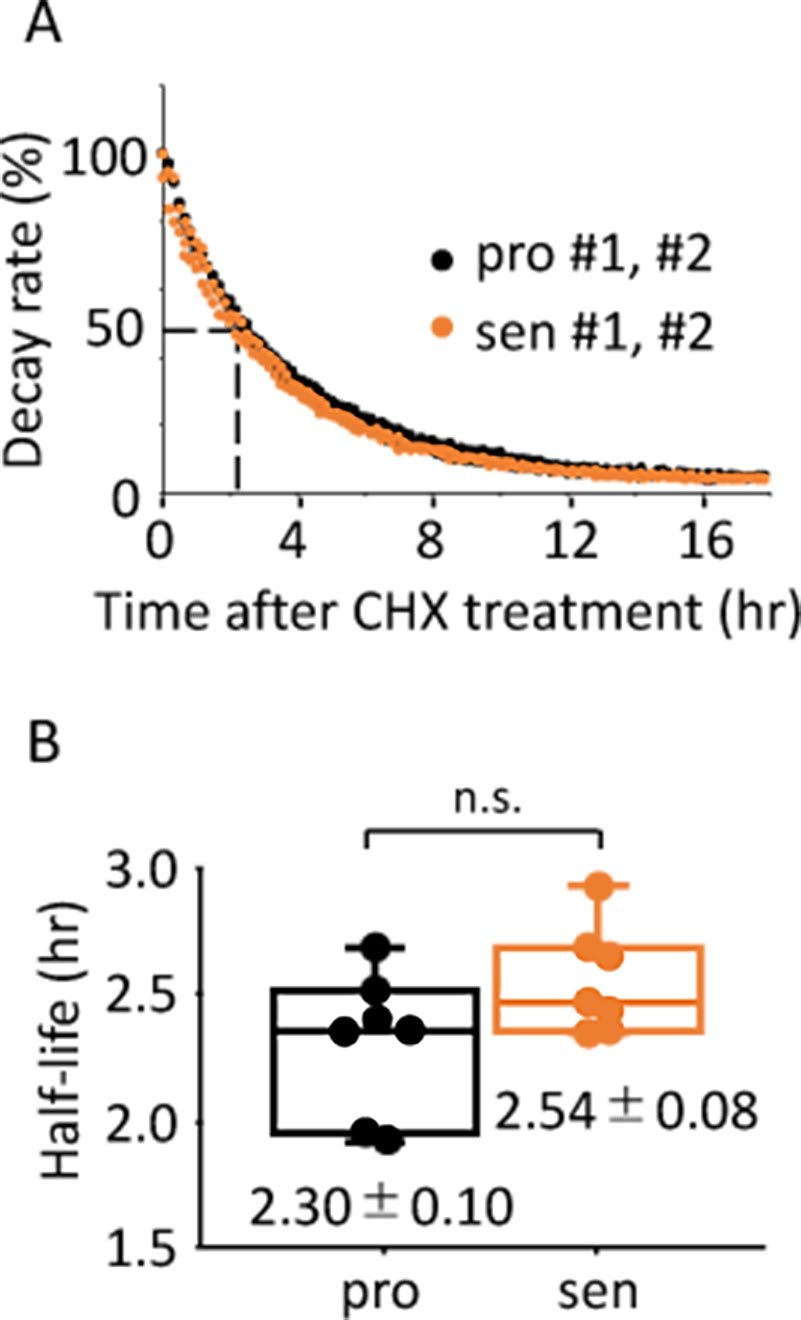
**Half-life of luciferase activity is comparable between the proliferative and senescent cells.** (**A**) Representative results of activity decay of luciferase in the proliferative (pro) and senescent (sen) cells are shown. Two independent experiments were performed. (**B**) Half-life of luciferase activity in the proliferative and senescent cells were quantified. Values are mean ± SEM. *p* = 0.09; n.s., not significant; n=7, by Student’s two-tailed *t* test.

Various cellular metabolisms/homeostasis are altered in senescent cells [50], for example, energy production in senescent fibroblast cells shifts to glycolysis with little to no change in oxidative phosphorylation [51]; while protein homeostasis through both ubiquitin proteasome pathway and autophagy is also impaired [2,52,53]. These findings raised the possibility that altered metabolisms/homeostasis in the senescent cells might have effects on luciferase activity, which is independent from the circadian clock. To address this possibility, the half-life of luciferase activity in the proliferative and senescent cells were monitored by performing a real-time luciferase monitoring assay (Fig.5A). Similar to the previous report [54], half-life of luciferase activity in the proliferative cells was 2.30 ± 0.10 hr, which was comparable to that in the senescent cells, 2.54 ± 0.08 hr (Fig.5B). This result showed that the senescent TIG-3 cells have no effect of altered metabolisms/homeostasis on the half-life of luciferase activity, demonstrating that circadian clock-independent, senescence-induced unknown metabolic alterations would not affect the circadian oscillation patterns monitored by luciferase intensity.

Taking all results in Figs.3-5, we concluded that a longer circadian period and delayed peak-time elicited by Dex are due to the altered oscillator of the circadian clock in senescent cells.

### Alteration to features of circadian clock in the senescent cells is a general hallmark of senescence of the circadian clock

**Figure 6:**
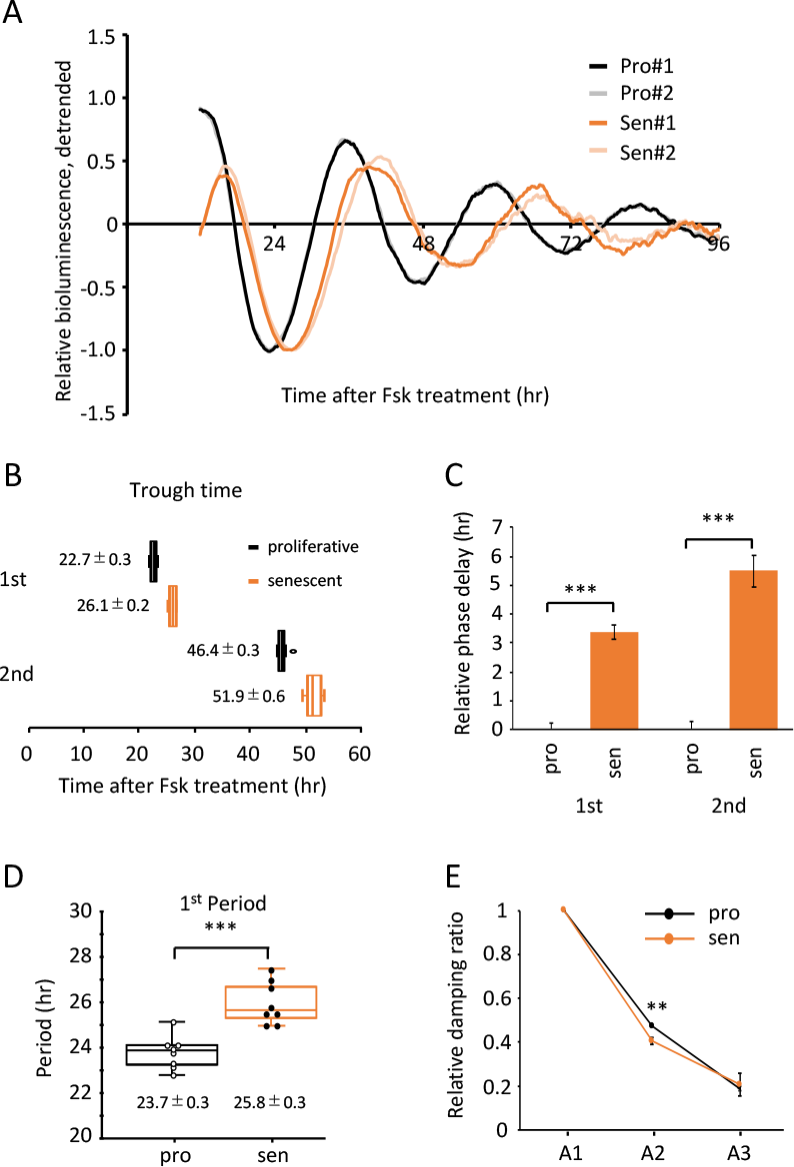
**Alteration of circadian clock in the senescent cells was also observed by a Fsk-induced entrainment.** (**A**) Relative oscillation patterns of luciferase in the proliferative (pro) and senescent (sen) cells entrained by Fsk were monitored by using a real-time luciferase monitoring system. Lowest intensity of each sample was set to -1. (**B**) Box-whisker plots of trough-times are displayed, n=8. Values are mean ± SEM. (**C**) Relative trough-time differences were measured, n=8. (**D**) Box-whisker plots of period lengths in the proliferative (pro) and senescent (sen) cells are displayed, n=8. Values are mean ± SEM. (**E**) Relative damping ratio in the proliferative (pro) and the senescent (sen) cells are analyzed. Data are mean ± SEM. **p<0.01, ***p<0.001, compared to each of the proliferative cells by Student’s two-tailed *t* test.

To elucidate whether the findings shown in Fig.2 are Dex-synchronization specific or can be generalized, we further analyzed the trough-time difference and the period of Fsk-synchronized TIG-3 cells. Superimposed relative oscillation patterns of luciferase demonstrated that the oscillation patterns in the senescent cells seemed to be delayed compared to the proliferative cells (Fig.6A, B and S1), similar to the results synchronized by Dex (Fig.2A and B). Statistical analyses revealed that the 1^st^ and 2^nd^ trough-times of senescent cells were significantly delayed (Fig.6C). In addition, the 1^st^ period length, the duration between 1^st^ and 2^nd^ trough-times, was longer in the senescent cells (23.7 ± 0.3 hr vs 25.8 ± 0.3 hr; *p*-value, 1.50 x 10^-4^; Fig.6D). We also analyzed the peak-time differences and the period using 2^nd^ and 3^rd^ peak-times (Fig.S6). Consistent with trough-time differences, differences of 2^nd^ and 3^rd^ peak-time between the proliferative and senescent cells were 4.7 ± 0.3 and 7.1 ± 1.0 hr, respectively (Fig.S6B). Again, the 2^nd^ period, which is the duration between 2^nd^ and 3^rd^ peak-times, in the senescent cells also demonstrated a longer period compared to the proliferative cells (23.5 ± 0.4 hr vs 25.6 ± 0.8 hr; *p*-value, 0.01; Fig.S6C). Finally, cosiner analyses revealed that the period length in the senescent cells is longer than that in the proliferative cells (23.79 ± 0.05 hr vs 26.91 ± 0.27 hr; *p*-value, 1.52 x 10^-8^; Table 2), and the acrophase in the senescent cells is delayed compared to that in the proliferative cells (-227.13 ± 2.37 degree and -257.50 ± 3.12 degree; *p*-value, 1.99 x 10^-6^; Table 2). Consistent with the result of Dex-induced entrainment, we again found that the damping ratios between the proliferative and senescent TIG-3 cells were comparable (Fig.6E).

**Table 2:** Cosinor Analysis of Experiments in Figure 6.

	**Period (hr)**	**acrophase (degree)**
**proliferative**	23.79 +/- 0.05	-227.13 +/- 2.37
**senescent**	26.91 +/- 0.27	-257.50 +/- 3.12
***p* value**	1.52 x 10^-8^	1.99 x 10^-6^

Student’s two-tailed *t* test were performed, n=8.

Taking all results together, we propose that the alteration to features of circadian clock in the senescent cells is not a Dex-synchronization specific phenomenon, but rather the general hallmark of senescence of the oscillator of circadian clock.

### Acute response and circadian expression profiles of endogenous clock genes are attenuated in the senescent cells

**Figure 7:**
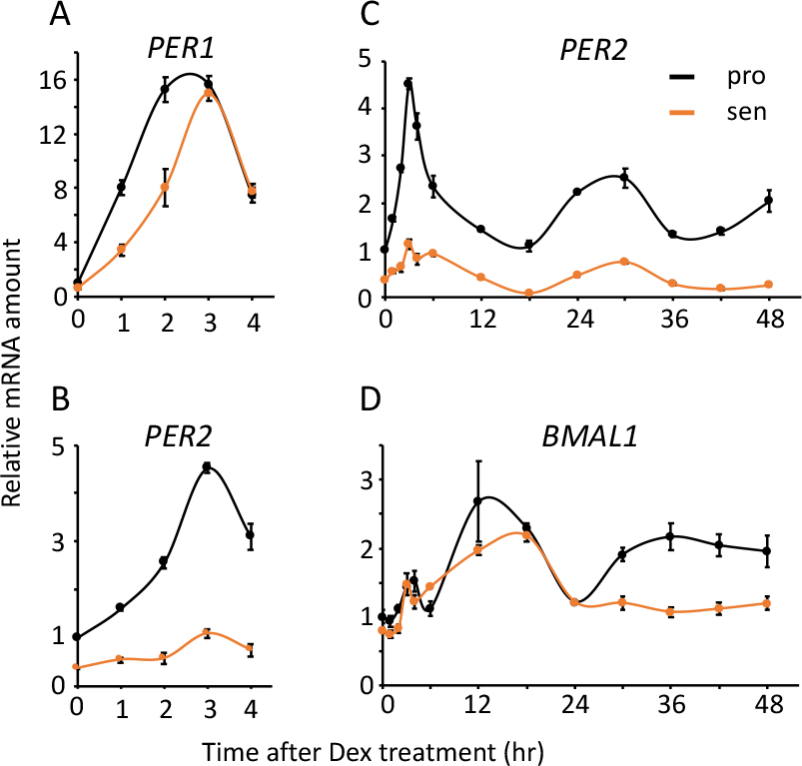
**Acute and circadian profiles of endogenous circadian genes were altered in the senescent cells.** Acute responses of *PER1* (**A**) and *PER2* (**B**), and circadian profiles of *PER2* (**C**) and *BMAL1* (**D**) induced by Dex were quantified. Each sample was normalized by 18S rRNA. Time 0 of proliferative cells were set to 1 for each gene.

Finally, we addressed expression profiles of endogenous circadian clock genes in TIG-3 cells. We first investigated the acute response of clock genes by Dex treatment. Although *PER1* and *PER2* are known to possess glucocorticoid response elements (GRE) in their promoters [48,49], their acute responses to Dex treatment in the senescent cells were different from those in the proliferative cells. Acute response of *PER1* was delayed, the peak-time was 3 hr after Dex treatment in the senescent cells, although the peak-time was approximately 2.5 hr in the proliferative cells (Fig. 7A). In contrast, acute response of *PER2* was severely weakened in the senescent cells (Fig.7B). Distinct responses of *PER1* and *PER2* were also observed by Fsk treatment (Fig.S7). Interestingly, acute responses of other clock genes, which have not been reported to possess GRE on their promoters, were grouped into two categories; one was *BMAL1* and *CRY2*, which showed similar expression levels and responses between the proliferative and the senescent cells (Fig.S8), the other was *CRY1* and *REV-ERBA*, which showed similar responses with different expression levels (Fig.S8). We further monitored circadian expression profiles of the endogenous clock genes in the proliferative and senescent cells. Expression levels of *PER2, CRY1* and *REV-ERBA* mRNAs in the senescent cells decreased to less than half compared to the proliferative cells throughout the time course, and amplitudes of these genes were attenuated (Fig.7C and Fig.S9). On the other hand, expression levels and oscillation patterns of *BMAL1, PER1* and *CRY2* were similar or moderately weakened in the senescent cells (Fig.7D and Fig.S9). In summary, we revealed that in the senescent cells the acute response of *PER1* and *PER2* are altered, which may correspond to the following delayed-phase, and *PER2*, *CRY1* and *REV-ERBA* demonstrate attenuated expressions and oscillation, which may give rise to the extension of circadian period in the senescent cells.

## DISCUSSION

Aging has been identified as one of the risk factors linked to the alteration of circadian physiology and behavior [24–28]. On the other hand, disruption of circadian clock has been recognized to bring about many physiological abnormalities, leading to a wide variety of age-related diseases and premature aging [16,17]. These pieces of information demonstrate that the mechanisms of aging and circadian clock are intimately regulated by each other at the animal level. However, little is known about their mutual regulations at the cellular level. Intriguingly, we have recently reported that the primary fibroblast cells derived from *Bmal1^-/-^* mouse embryo, in which circadian clock is completely disrupted, do not demonstrate the acceleration of replicative and stress-induced senescence [29], suggesting that circadian clock does not control cell-autonomous cellular senescence. Therefore, the current study addressed the reverse question, i.e. whether cellular senescence could affect the circadian clock, and provided evidence for the first time that senescent cells demonstrate a prolonged period and delayed peak-time (Figs. 2 and 6), which was successfully achieved due to the performance of high time-resolution (10 min) real-time luciferase monitoring system. Importantly, these findings were observed by two different entrainment factors, Dex and Fsk, and senescence had no effect on input pathways to the oscillator (Fig.3). Our current study, therefore, indicate that the altered circadian clock with a prolonged period and delayed peak-time is the general hallmark of senescence of the “oscillator” of circadian clock. Furthermore, we uncovered at the cellular level that aging could affect the circadian clock, but not *vice versa*.

In the present study, luciferase intensity in the senescent cells was much higher than that in the proliferative cells (Fig.3A, S1 and S4). Since endogenous *BMAL1* mRNA levels, promoter of which drives *luciferase* in this study, were comparable between the proliferative and senescent cells (Fig.4A and 7D), the high luciferase intensity could be due to high infection efficiency in the senescent cells. We used VSV-G pseudotype lentivirus in this study, and cellular receptor for the envelope protein VSV-G has been known to be membrane lipids [55]. Some literature has reported that certain lipid families accumulate during replicative senescence [56,57], suggesting that the accumulation of lipids may contribute to the high susceptibility in the senescent TIG-3 cells. Importantly, we showed that infection efficiency has no effect on the oscillation pattern of luciferase (Fig.4B).

Nakamura *et al.* performed well-designed *ex vivo* SCN tissue study [40]. In that paper, they demonstrated that i) peak phase was delayed in the aged SCN, ii) phase differences gradually increased, and iii) period length in the aged SCN was longer than that in young SCN; all of which are consistent with our current study. In addition, it has been reported that aged animals display more variability in the time of activity onset, and require more days to be re-entrained following new light-dark schedule than young animals [26–28], which are similar to the current results in that the variability in trough-time was wider and the trough-time was delayed in the synchronized senescent cells (Fig.2B-C and 6B-C). Our novel findings at the cellular level are consistent with the phenotypes at the tissue and animal levels, indicating that the alternation of circadian clock at tissue and animal levels is caused by the alterations of the oscillator of circadian clock in the cells, although we cannot deny the possibility that aged animals may have altered cell to cell and/or organ to organ communications for the circadian clock.

It is well known that photic stimulation induces rapid expressions of *per1* and *per2* in the SCN to give rise to the light-induced entrainment [58,59]. Light-induced rapid inductions of *per1* and *per2* in the SCN are also known to be attenuated with aging [60,61]. Consistent with these reports, we demonstrated in the current study that *PER1* and *PER2* rapid induction by Dex-induced entrainment is attenuated in the senescent TIG-3 cells. However, their profiles are not same; *PER1* induction is delayed, but its expression level is comparable to that in the proliferative cells, on the other hand, *PER2* induction is almost completely repressed. This difference suggests that gene regulations of *PER1* and *PER2* are distinct. Epigenetic changes such as DNA methylation, which has been proposed to play a central role in senescence and aging [2], is one candidate regulation. Furthermore, our finding suggests that not only acute response of *PER2*, but circadian expression profiles of *PER2*, *CRY1* and *REV-ERBA* are also epigenetically modified. Further investigations will reveal molecular mechanisms of how the circadian gene expressions are regulated in the senescent cells.

Understanding circadian biology of aging in humans is a pressing challenge for the global aging society. Two groups have developed virus-based circadian reporter and succeeded to characterize circadian rhythms in human skin biopsy or human hair follicle tissue [62,63] for investigating the impact of aging on circadian clock, instead of using cultured primary cells such as the current study. Both reports demonstrated that the circadian period lengths have no difference between young and elder subjects. This discrepancy in their and our results is probably due to reasons as follows; samples from human subjects have the inter-individual differences of circadian period length, which were much more variable than different samples of the same individual. Moreover, samples from human elder subjects may have more inter-individual variations of the ratio of senescent and non-senescent cells than those from our current study. Although their assay systems reflect more physiological circadian characteristics of aging, our assay system can control the genetic/environmental backgrounds and the ratio of senescent cells. We believe that our current study demonstrated the possibility of primary cells for studying *in vitro* circadian biology of aging. Further investigations using both primary cells and biopsy samples will be needed to assess in-depth mechanisms of aging in circadian clock.

In conclusion, in our current study at the cellular level, we revealed for the first time that replicative senescent cells demonstrate the delayed peak-time and prolonged period compared to the proliferative cells, uncovering that the circadian clock is altered with the establishment of cellular senescence. These findings are consistent with the prior studies at tissue and animal levels. Although, in this study, we have not demonstrated any molecular mechanisms of how aging alters the circadian clock, which is still largely unknown, we propose that investigation at the cellular level is a powerful and useful approach to dissect the molecular mechanisms of how aging alters the circadian clock. Further investigations using primary cells could shed light on the molecular mechanisms of aging in circadian clock, and perhaps help to promote healthy aging and longevity in the elderly.

## MATERIALS AND METHODS

### Cell culture

TIG-3 cells, which were kindly provided by Drs. T. Takumi and T. Akagi, were grown in Dulbecco's Modified Eagle Medium (4.5 g/L glucose) (Nacalai Tesque, Kyoto, Japan) supplemented with 10% fetal bovine serum (biowest, Nuaillé, France) and 10 U/ml penicillin/10 µg/ml streptomycin (Nacalai Tesque, Kyoto, Japan) at 37 °C and 5% CO_2_.

### Cell growth assay

TIG-3 cells were cultured in triplicates in 10 cm dishes at seeding density = 5x10^5^ cells/10 cm dish. Upon reaching semi-confluence; every 3 days till passage 34 and 4 days or more from passage 35, the cells were trypsinized, counted and seeded again at 5x10^5^ cells/10cm dish. This process was repeated until the cells stopped proliferation and three independent assays were performed. To calculate the Population Doubling Level (PDL), the following formula was used: PDL=3.32log(N_harvest_/N_seeding_), where N_seeding_ is the seeding cell number and N_harvest_ is the cell number at harvest when cells reach semi-confluence. Cumulative PDL (cPDL) of a particular passage number is the accumulation of all the previous PDL values, including that of the passage being measured. In this study cells are defined as senescent when the PDL value of the cells is below 1.

### Senescence associated β-galactosidase (SA-β-Gal) activity

Cells were fixed with 2% formaldehyde and 0.2% glutaraldehyde in H_2_O for 5 min and incubated at 37 °C without CO_2_ for 16 hr in X-Gal staining solution (40 mM sodium citrate (pH 6.0), 5 mM potassium ferrocyanide, 5 mM potassium ferricyanide, 1 mg/ml 5-bromo-4-chloro-3-indolyl-β-D-galactopyranoside (X-gal), 150 mM sodium chloride, and 2 mM magnesium chloride). 200 cells were counted under a bright-field microscope for each sample.

### RNA extraction and qPCR

Total RNA from the TIG-3 cells were extracted using Sepasol RNA-I Super G (Nacalai Tesque, Kyoto, Japan). Total RNA was reverse-transcribed by using SuperScriptII Reverse Transcriptase (Invitrogen, Carlsbad, CA, USA) with random primers in accordance with the manufacturer’s instructions. Quantitative PCR was performed in the presence of KAPA SYBR FAST Universal 2X qPCR Master Mix (Nippon Genetics, Tokyo, Japan) on the Light Cycler 480 (Roche, Basel, Switzerland) under the following conditions: 95 °C for 3 min, and 40 cycles at 95 °C for 10 sec, 60 °C for 20 sec and 72 °C for 1 sec. The sequences of the forward and reverse primers are as follows. *p16^INK4a^* FW; ACC AGA GGC AGT AAC CAT GC, *p16^INK4a^* RV; GGA CCT TCG GTG ACT GAT GA. *p21^CIP^* FW; AGC ATG ACA GAT TTC TAC CAC TCC, *p21^CIP^* RV; GCA GAA GAT GTA GAG CGG GC. *PER1* FW; GAG AGC AGC AAG AGC ACA AA, *PER1* RV; AGC TCT CGA AGT GCT GTC AT. *PER2* FW;TCC AGT GGA CAT GAG ACC AA, *PER2* RV; CGC TAC TGC AGC CAC TTG TA. *CRY1* FW; CAG CAG TGG AAG TTG CTC TC, *CRY1* RV; CTA GGA CGT TTC CCA CCA CT. *CRY2* FW; GGG AGG AGA GAC AGA AGC TC, *CRY2* RV; GAG GGA GTT GGC GTT CAT TC. *REV-ERBA* FW; GAC ATG ACG ACC CTG GAC TC, *REV-ERBA* RV; GCT GCC ATT GGA GTT GTC AC. *BMAL1* FW; CAT GCA ACG CAA TGT CCA G, *BMAL1* RV; GTG TAT GGA TTG GTG GCA CCT. The primer set of 18S rRNA was described preciously [35].

### Lentivirus production and infection to TIG-3 cells

HEK293T cells were seeded at 4x10^6^ cells in 10 cm dish. After 24 hr of seeding, when the cells reached 80-90% confluency, transfection was performed as follows: two 1.5 mL tubes were taken and 200 μL of Opti-MEM (WAKO, Osaka, Japan) was put into each tube. Into one tube, 9 μg of psPAX2, 6 μg of pMD2.G and 30 μL of 1 mg/ml polyethylenimine (PEI) (Polysciences, Warrington, PA, USA) were added. Into the other tube, 14 μg of pLV6-Bmal1-luc and 28 μL of PEI were added. Then the components of each tube were mixed by repeated inversion and then incubated at room temperature for 15 minutes. The contents of each tube were then added to the HEK293T cell dish. After 24 hr, the medium was removed from the cells, washed with PBS once and 5 mL of fresh medium was added. 24 hr later, the viral supernatant was collected and stored in 4°C, and 5 mL of fresh medium was added to the dish again. After 24 hr the viral supernatant was collected and pooled with the viral supernatant of the previous day. The total volume of viral supernatant was centrifuged at 167.7 x g at room temperature for 5 min to get rid of any cellular debris. The supernatant was further filtered through a 0.2 μm filter to remove any remaining debris of HEK293T cells. The filtered viral suspension was then centrifuged at 8,000 x g at 4°C for 4 hr to concentrate the lentivirus particles. After 4 hr, the supernatant was removed, and the lentivirus pellet was resuspended in 2 mL of medium to obtain the viral suspension.

For infection of the target TIG-3 cells, which had been seeded on the previous day at 1.5x10^5^ cells/35 mm dish, the culture medium was replaced with the viral suspension supplemented with protamine sulfate. 24 hr later the cells were washed with PBS once and 2 mL of fresh medium was added. The cells were then cultured for an additional 48 hr.

### Synchronization of circadian clock in TIG-3 cells for Real-time luciferase monitoring assay and qPCR

48 hr after medium change of the infected TIG-3 cells, the medium was replaced with fresh 2 mL medium containing 0.1 μM dexamethasone (Dex; Nacalai Tesque, Japan) or 20 μM forskolin (Fsk; Nacalai Tesque, Japan), and incubated for 1 hr at 37°C 5% CO_2_ for the entrainment of the circadian clock. Afterwards, the medium was removed, cells washed with PBS twice and medium containing 100 μM D-luciferin (Nacalai Tesque, Kyoto, Japan) was added. The cells were placed in the KronosDio luminometer (ATTO, Tokyo, Japan) and bioluminescence of the cells were measured for 5 days continuously. After 5 days, raw data were collected and in order to observe the circadian gene expression pattern more precisely, detrended data were also retrieved from the KronosDio software.

For the evaluation of endogenous circadian expression profiles, TIG-3 cells at confluent conditions were treated with 0.1 μM dexamethasone for 1 hr. Then the medium was replaced with normal culture medium and cells were cultured until indicated time points. RNA extraction, cDNA synthesis and qPCR were described above.

### Cosinor analysis

To perform Cosinor analyses, data of three-day detrended samples from real-time luciferase monitoring assay were used. Cosinor software was kindly provided by Dr. R Refinetti [42].

### Half-life of luciferase activity

*Bmal1-luc*-infected TIG-3 cells were treated with 50 μg/ml cycloheximide, then cells were set to the real-time luciferase monitoring system. Half-life of luciferase activity was evaluated at the time when decay rate became 50%.

### Statistics

Values are reported as mean ± SEM. Statistical differences were determined by a Student's two-tailed *t* test. Statistical significance is displayed as * (p < 0.05), ** (p < 0.01), or *** (p < 0.001).

## Supplementary Material

Supplementary Figures
