# Supplementary material for: Replicative senescent human cells possess altered circadian clocks with a prolonged period and delayed peak-time
Source: Aging (Albany NY). 2019 Feb 9;11(3):950–73. doi: 10.18632/aging.101794 (PMC6382424; doi:10.18632/aging.101794)
Supplement: Supplementary Figures [file aging-11-101794-s001.pdf]

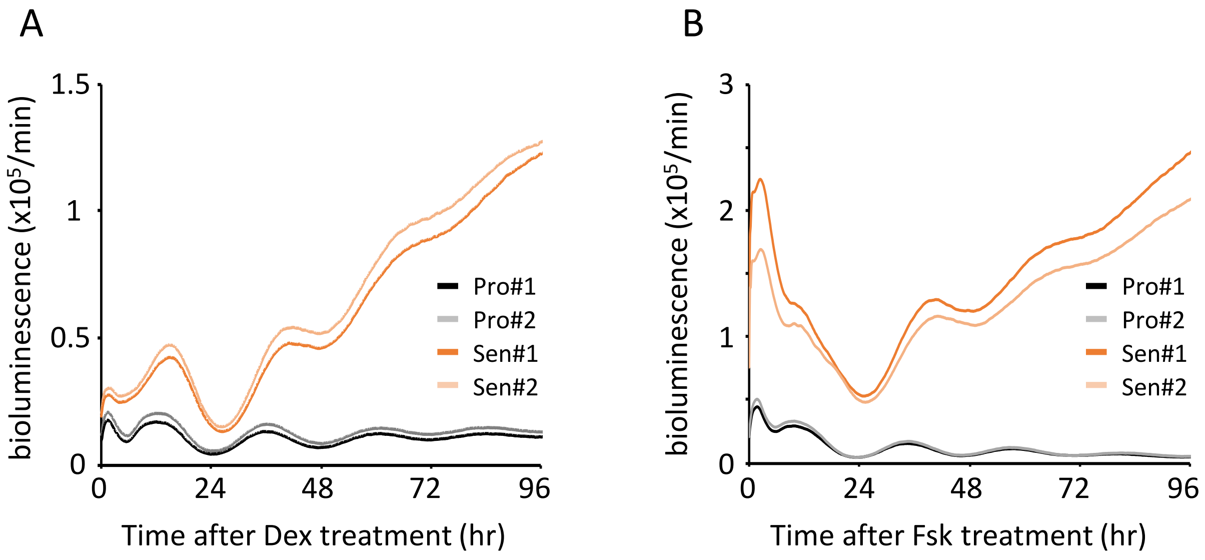

Figure S1. Oscillation patterns of luciferase in the proliferative and senescent cells. Raw data of Fig.2A (**A**) and 6A (**B**) are shown. Black and orange lines depict the results in proliferative and senescent cells, respectively.

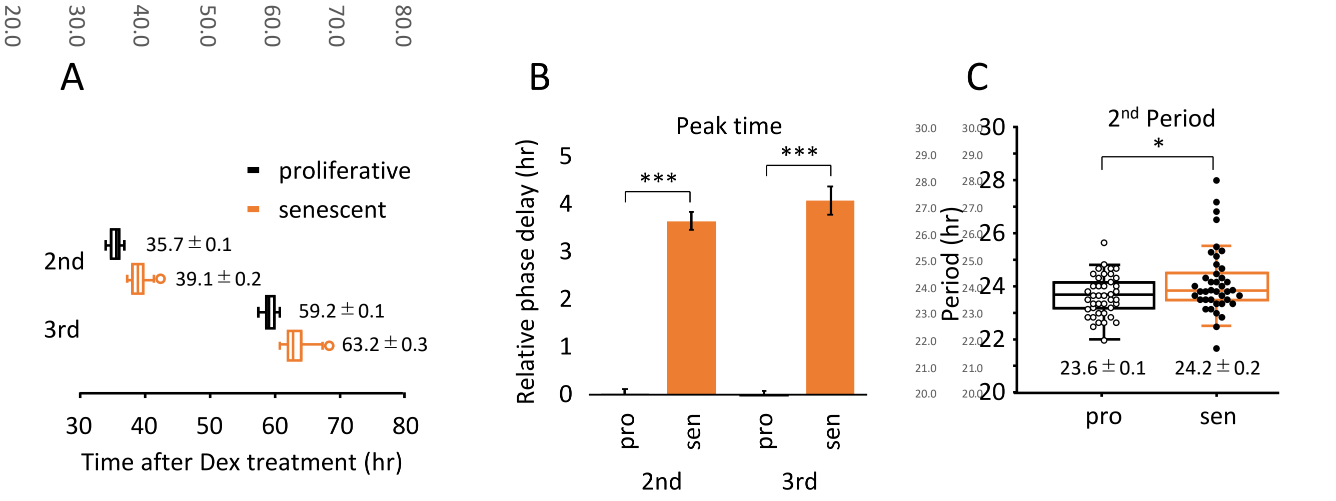

Figure S2. Alteration of circadian clock in the senescent cells was observed by a "Dex"-induced entrainment. (**A**) Box-whisker plots of peak-times are displayed, n=41. Values are mean ± SEM. (**B**) Relative peak-time differences were measured, n=41. (**C**) Box-whisker plots of period lengths in the proliferative (pro) and senescent (sen) cells are displayed, n=41. Values are mean ± SEM. *p<0.05, ***p<0.001, compared to each of the proliferative cells by Student's two-tailed *t* test, n=41.

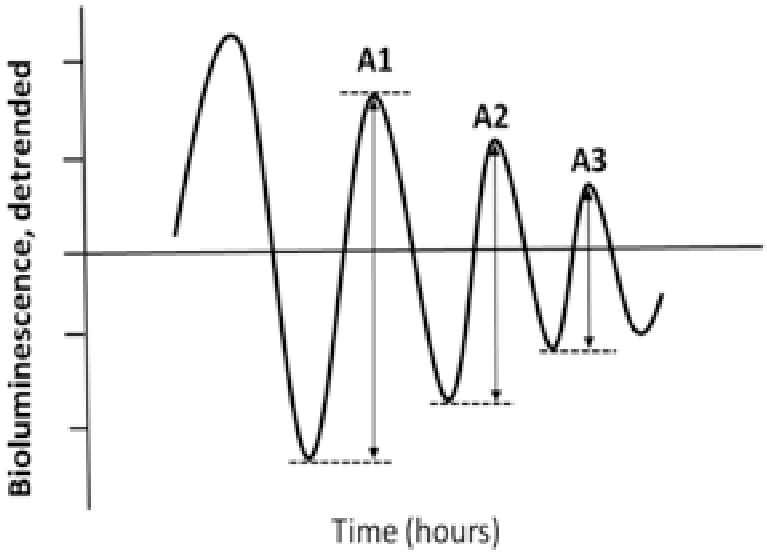

Figure S3. Scheme of how damping ratio are measured in this study. A1, A2, and A3, between top to nadir of each oscillation, were determined. Range of the 1st oscillation (A1) was set to 1 and the damping ratios of A2 and A3 were calculated.

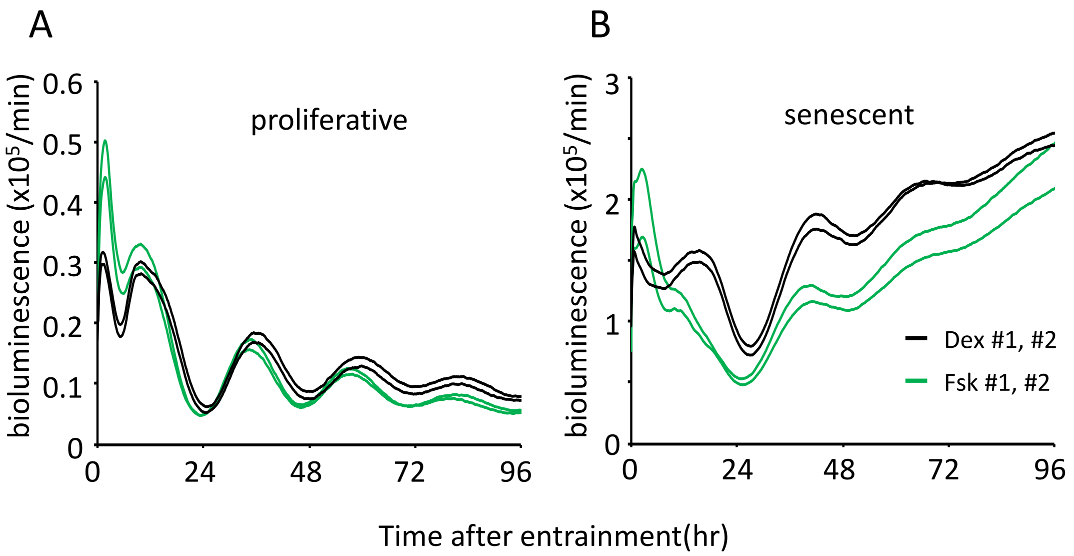

Figure S4. Oscillation patterns of luciferase in the proliferative and senescent cells. Raw data of Fig.3A (A) and B (B) are shown. Black and green lines depict the results of Dex- and Fsk-treated cells, respectively.

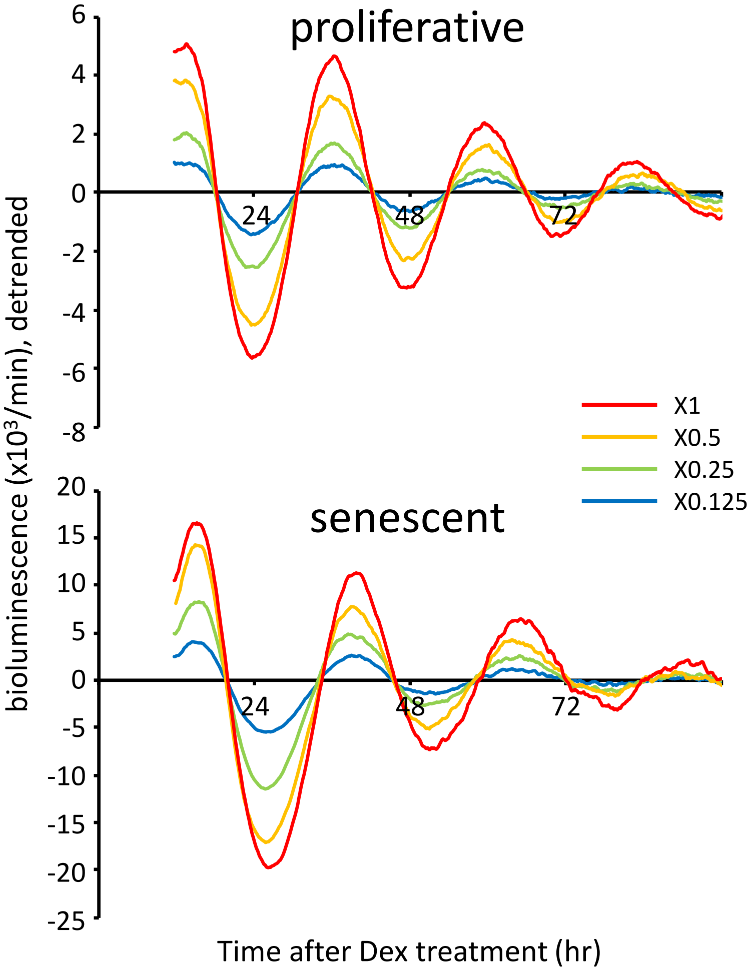

Figure S5. Effects of virus dosage on circadian clocks in the proliferative and the senescent cells. Oscillation patterns of luciferase in serially diluted virus-infected TIG-3 cells are shown. Two independent experiments were performed.

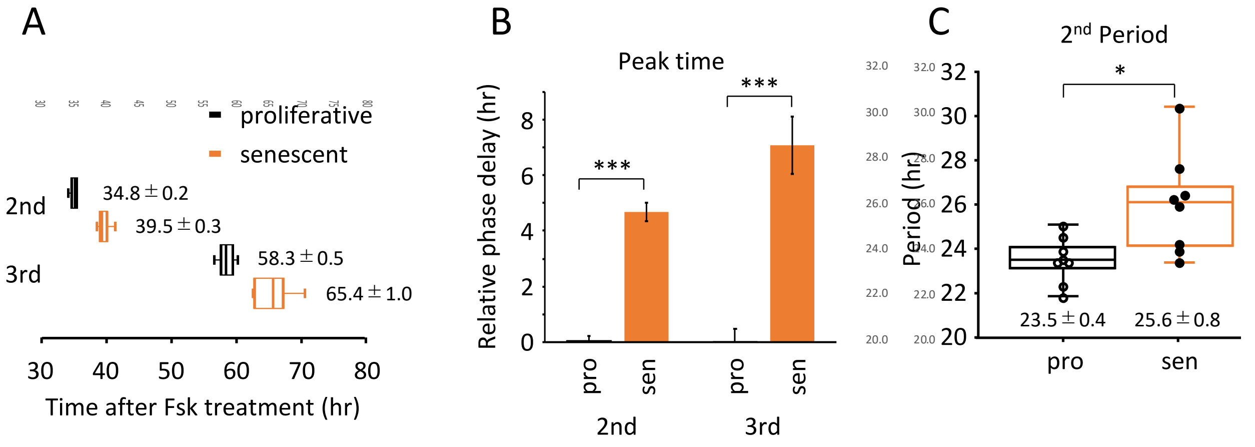

Figure S6. Alteration of circadian clock in the senescent cells was observed by a "Fsk"-induced entrainment. (**A**) Box-whisker plots of peak-times are displayed, n=8. Values are mean ± SEM. (**B**) Relative peak-time differences were measured, n=8. (**C**) Box-whisker plots of period lengths in the proliferative (pro) and senescent (sen) cells are displayed, n=8. Values are mean ± SEM. *p<0.05, ***p<0.001, compared to each of the proliferative cells by Student's two-tailed *t* test, n=8.

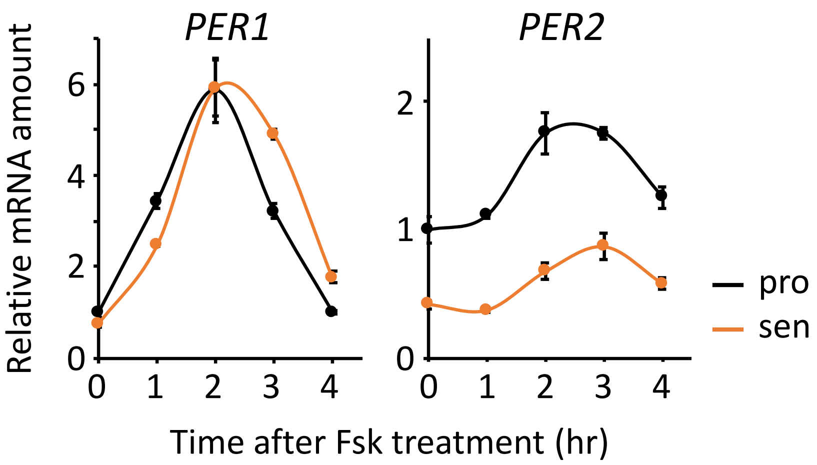

Figure S7. Acute responses of clock genes were quantified. Acute responses of *PER1* and *PER2* induced by Fsk were quantified by qPCR. Each sample was normalized by 18S rRNA. Time 0 of proliferative cells were set to 1 for each gene.

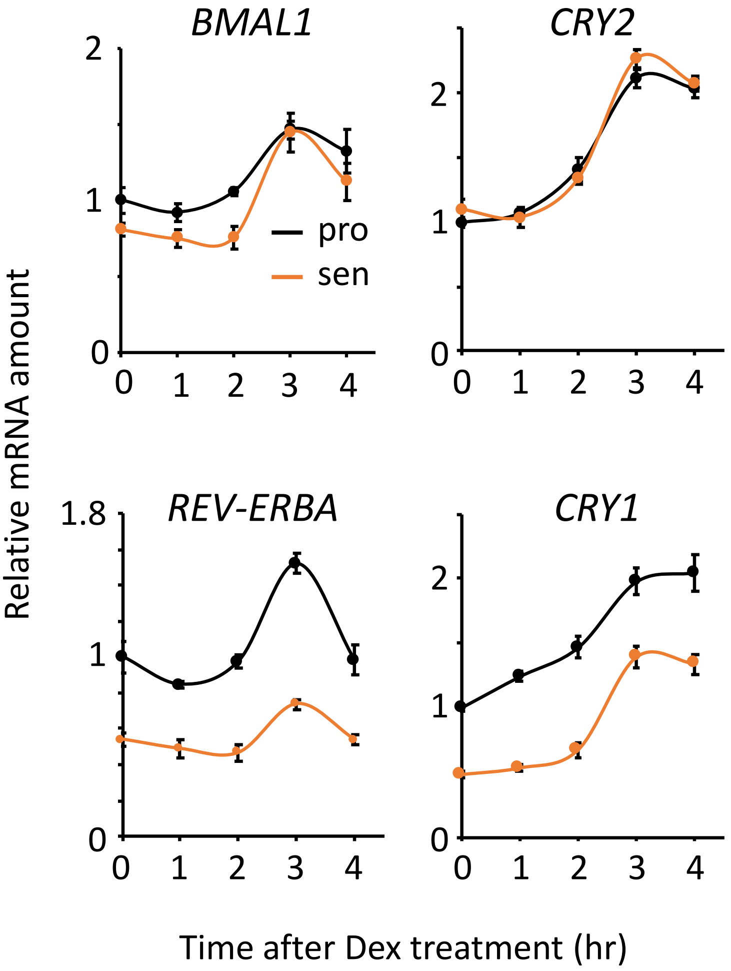

Figure S8. Acute responses of clock genes were quantified. Acute responses of *CRY2*, *REV-REBA* and *CRY1* induced by Dex were quantified by qPCR. Each sample was normalized by 18S rRNA. Time 0 of proliferative cells were set to 1 for each gene.

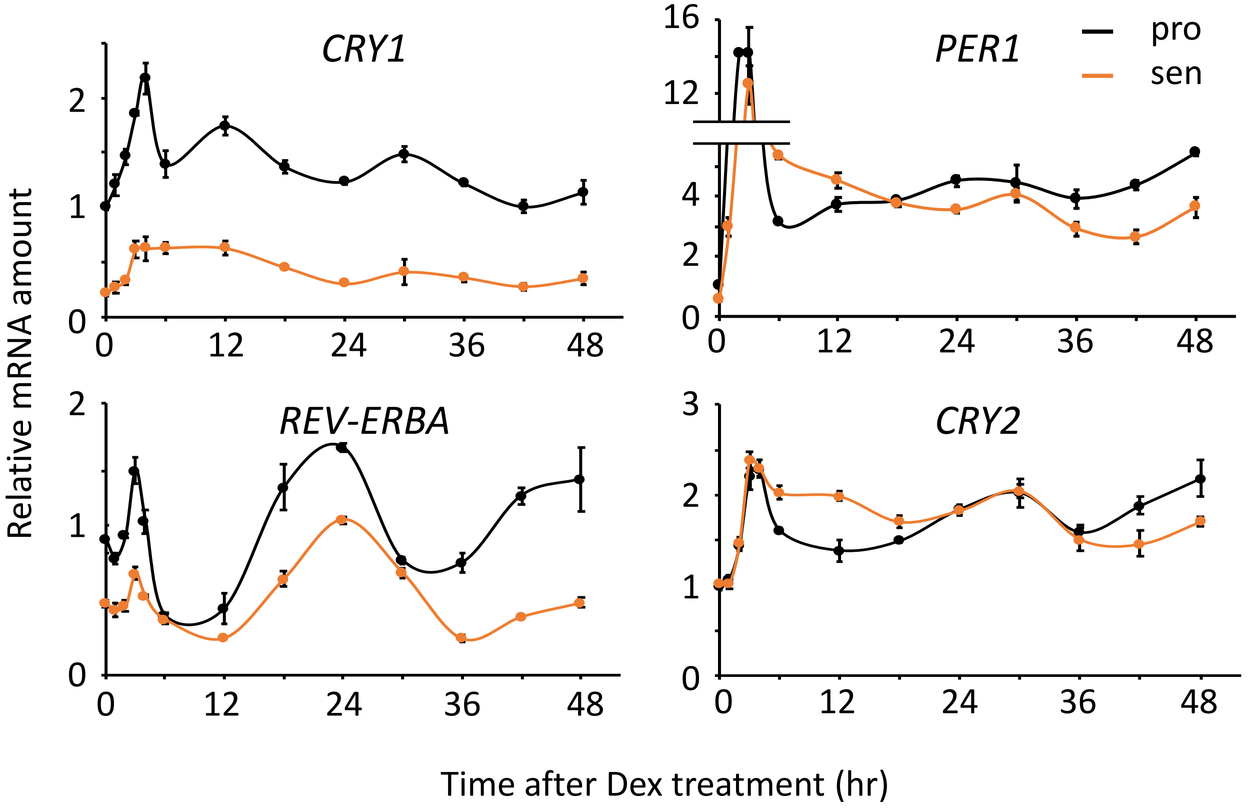

Figure S9. Circadian expression profiles of endogenous circadian genes were quantified. Circadian expression profiles of *CRY1, REV-ERBA, PER1* and CRY2 were quantified by qPCR. Each sample was normalized by 18S rRNA. Time 0 of proliferative cells were set to 1 for each gene.
